# Traditional healers can help facilitate HIV serostatus disclosure: results from a qualitative study in rural Uganda

**DOI:** 10.1002/jia2.70031

**Published:** 2025-09-25

**Authors:** Srija Gogineni, Gabriel Nuwagaba, Misha Hooda, Sylvia Natukunda, Constance Birungi, William Bugeza, Maureen Tushabe, Denis Nansera, Winnie Muyindike, Carolyn Marie Audet, Juliet Mwanga‐Amumpaire, Radhika Sundararajan

**Affiliations:** ^1^ Center for Global Health Weill Cornell Medicine New York New York USA; ^2^ Mbarara University of Science and Technology Mbarara Uganda; ^3^ Immune Suppression Syndrome Clinic Mbarara Regional Referral Hospital Mbarara Uganda; ^4^ Department of Health Policy & Institute for Global Health Vanderbilt University Nashville Tennessee USA; ^5^ Department of Emergency Medicine Weill Cornell Medicine New York New York USA

**Keywords:** HIV serostatus disclosure, traditional healers, HIV‐related stigma, psychosocial support, PLWH (people living with HIV), community‐based interventions

## Abstract

**Introduction:**

In Uganda, HIV‐related stigma and discrimination remain major barriers to HIV care engagement and serostatus disclosure. While serostatus disclosure can improve access to, engagement with and retention in HIV care, many people living with HIV (PLWH) hesitate to disclose due to fear of negative consequences. Traditional healers (THs) are trusted community members offering accessible and confidential psychosocial support. This study explores the role of THs in facilitating HIV status disclosure among PLWH disengaged from clinical care in southwestern Uganda.

**Methods:**

This qualitative sub‐study was nested within a cluster‐randomized trial evaluating the effectiveness of THs supporting PLWH to engage with HIV care in southwestern Uganda. In‐depth semi‐structured individual interviews were conducted with 22 healers (14 female) and 16 PLWH (10 female) from August 2023 to June 2024. Interviews explored experiences with HIV care and healer‐facilitated support to engage with and remain in HIV care. Data was analysed thematically, with particular attention to serostatus disclosure practices.

**Results:**

Four key themes emerged: (1) PLWH, who receive care from TH practices, preferred THs over healthcare workers to disclose their HIV serostatus due to perceived trust, confidentiality and personalized care; (2) HIV‐related stigma and fear of domestic violence hindered disclosure within families, but disclosure to healers offered a safer alternative; (3) in some cases, THs were the first individuals to whom PLWH disclosed their status; and (4) THs actively encouraged and facilitated serostatus disclosure by PLWH to family members, offering guidance and mediating difficult conversations. These findings highlight the critical role of THs in reducing barriers to disclosure and fostering supportive networks to improve the quality of life for PLWH.

**Conclusions:**

THs provide a culturally sensitive and trusted avenue for HIV status disclosure in rural Uganda. Their unique position within the community allows them to address stigma, build trust and facilitate safe disclosure practices. Integrating healers into HIV care through training and collaboration with formal healthcare systems could enhance linkage, adherence, retention and overall care outcomes for PLWH. Future research should explore scalable models to leverage the positive influence and potential of THs to improve HIV care delivery.

## 1 INTRODUCTION

African countries have the greatest burden of HIV globally, with nearly three‐quarters of all people living with HIV (PLWH) residing there [1, 2]. Despite heavy investment from PEPFAR and other multinational aid organizations, Uganda still has one of the highest rates of HIV in Africa [2]. HIV‐related stigma and discrimination remain a central barrier to ending the HIV epidemic [2, 3, 4]. There are various forms of stigma: internalized stigma occurs when individuals perceive themselves as being of lower worth [5], and anticipated stigma occurs when PLWH fear experiencing discrimination. Discrimination is when individuals are treated unfairly or suffer abuse due to their HIV status. High levels of internalized and anticipated HIV‐related stigma negatively impact the entire HIV care continuum, from delay in care seeking to reducing anti‐retroviral therapy (ART) adherence and retention in care [6, 7, 8, 9], undermining efforts to end HIV transmission by disrupting the steps needed to achieve HIV viral suppression [2].

HIV serostatus disclosure can improve all steps of the HIV continuum of care, from entry into care to ART adherence and retention in care [10, 11, 12]. Disclosure can help increase social support and improve self‐efficacy, which can subsequently lead to treatment adherence [13, 14]. Conversely, lack of disclosure to family members and partners has been shown to negatively impact adherence to anti‐retroviral medication and HIV treatment [15, 16, 17]. However, PLWH are often hesitant to disclose their serostatus due to fear of stigma and discrimination [18, 19, 20]. Data from one survey conducted in Uganda indicate that nearly two‐third of PLWH avoid healthcare facilities out of concern for facing discrimination and inadvertent status disclosure by healthcare workers. Currently, PLWH receive counselling on status disclosure as part of routine HIV care received in clinical settings; unfortunately, it is challenging for healthcare workers to facilitate status disclosure because clinics often lack privacy, and healthcare workers are unable to devote time and attention to PLWH to address their emotional concerns [13].

Our research in rural Uganda has shown that traditional healers (THs) are important community partners in HIV care, as many adults preferentially receive care from THs in their communities [21]. In the Ankole region of southwestern Uganda, THs are central figures in community health. They are community‐recognized practitioners who provide healthcare using indigenous knowledge, including herbal medicine, spiritual practices, and culturally rooted approaches to physical, mental and social wellbeing. PLWH utilize THs because of longitudinal trusting relationships, easy accessibility compared to healthcare clinics, perceived effectiveness of traditional remedies and underlying mistrust of biomedical care [22, 23]. Their roles are shaped by longstanding cultural beliefs and embedded trust within the community. These practitioners often maintain multi‐generational ties with families, which further strengthen their influence and relevance in matters of health, including HIV care.

We have shown that THs can effectively improve HIV testing uptake among African adults who otherwise would have sought testing at formal health facilities through the delivery of counselling and point‐of‐care HIV testing [21, 24]. While our prior work has shown that healers can facilitate HIV testing and support entry into HIV care, here we examine how THs support HIV serostatus disclosure, a critical component in the HIV care continuum. In our parent study, we are currently investigating whether Ugandan THs can improve engagement and retention in HIV care among rural PLWH after receiving training in providing social support and adherence counselling [22] (NCT 05943548). As part of the study, healers in the intervention arm first attended a joint 1‐day training session with the control group where healers were taught to provide pre‐HIV counselling and facilitate OraQuick Self‐Testing, followed by 2 additional days of instruction focused on delivering psychosocial support to PLWH using a tailored curriculum designed to complement routine HIV care [22, 25]. Following the training and study initiation, we conducted qualitative interviews with both THs and PLWH who were either newly diagnosed by the healer through a reactive self‐test result, or who had disengaged from HIV care. We examined if and how THs could specifically facilitate HIV status disclosure among rural Ugandans living with HIV. We explore whether THs provide a supportive environment for disclosure, guided in part by the HIV Positive Serostatus Disclosure Model [26, 27], which outlines the interpersonal and contextual conditions that support safe serostatus disclosure. This study fills an important gap by describing the specific strategies THs use to promote safe disclosure among PLWH, particularly among those disengaged from formal HIV care.

## 2 METHODS

### 2.1 Study design

This qualitative sub‐study was conducted as part of a cluster randomized hybrid type 1 effectiveness‐implementation trial involving THs in the Mbarara and Rwampara districts of southwestern Uganda. In the parent trial, we are evaluating the impact of TH‐delivered psychosocial support on ART adherence and retention in care. THs are randomly assigned in a 1:1 ratio to either an intervention or a control arm. THs randomized to the intervention arm first participated in a joint 1‐day training session with the control group before the trial began, followed by 2 additional days of training [22, 25]. These sessions focused on principles and strategies for facilitating HIV self‐testing and delivering psychosocial support to PLWH, using a TH‐tailored curriculum designed to complement routine HIV care [22, 25].

In the intervention arm, healers provide 12 months of psychosocial support to adults with unsuppressed HIV viral loads receiving care at their practices, while those in the control arm facilitated HIV self‐testing only in addition to their usual practices. We conducted baseline and 3‐month semi‐structured interviews with all THs assigned to the study intervention arm and a purposive sample of PLWH clients in the intervention arm after their first visit with THs. The data collection period was from August 2023 to November 2024. This qualitative study focused exclusively on the intervention arm interviews to examine the broad experiences of PLWH and THs participating in the adherence support intervention.

### 2.2 Study population and sampling

All THs randomized to the intervention study arm were invited to participate in qualitative interviews. PLWH participants were purposively sampled to ensure diverse representation based on gender, age, geographic location, and whether they were newly diagnosed with HIV at the healer or had disengaged from HIV clinical care. Participants were selected to capture a wide range of experiences with the intervention.

### 2.3 Data collection

Qualitative interviews lasted approximately 60 minutes and were conducted in private settings to ensure participant comfort and confidentiality. All interviews followed interview guides which were initially developed in English, translated into Runyankole (the local language), and then translated back to English to maintain fidelity of meaning. Interviews were conducted in Runyankole by trained members of the Ugandan qualitative research team, audio‐recorded, and later transcribed and translated into English. The English transcripts were reviewed against the audio recordings by the Qualitative Data Coordinator and the Ugandan Principal Investigator (both fluent in Runyankole and English) to ensure translation accuracy and data integrity.

### 2.4 Data analysis

We conducted a thematic analysis to identify key themes [28, 29]. Our initial coding framework used *a priori* codes focused on (1) PLWH experiences with HIV care (including testing, ART use and clinic visits); and (2) perceptions of the TH‐delivered intervention. Although our interview guides did not explicitly probe for disclosure experiences, the role of THs in supporting serostatus disclosure emerged as a cross‐cutting theme during early coding. After identifying this theme, we conducted a focused analysis across all transcripts to explore this phenomenon more deeply. Findings related to serostatus disclosure were identified across all baseline and 3‐month follow‐up interviews with THs, and all 16 baseline interviews with PLWH. While most PLWH disclosure quotes were from baseline interviews, 3‐month interviews with healers provided insight into how disclosure dynamics changed over time. The iterative nature of data collection allowed us to incorporate emerging codes into the codebook. Thematic saturation was reached when no new themes related to disclosure emerged from subsequent interviews. Saturation was monitored iteratively by the coding team during analysis, and we noted redundancy in disclosure‐related themes after coding approximately 80% of transcripts. Initial coding was done by SG, and codes were refined through discussion with authors GN and RS. Codes were then organized into overarching themes pertaining to HIV care, which included confidentiality, stigma and the provision of psychosocial support by THs.

Eligibility screening was conducted at the healer locations. All participants, including both healers and their clients, provided written informed consent in the local language (Runyankole) to a Ugandan research assistant involved in the study. A Data Safety and Monitoring Committee approved the consent forms and study protocol prior to the launch of this study. Ethical approval for this study was obtained by the institutional review boards of Weill Cornell Medicine (New York, NY, USA; 22–09025268), Mbarara University of Science and Technology (Mbarara, Uganda; MUST‐2022‐646), and approved by the Ugandan National Council of Science and Technology (HS2780ES).

## 3 RESULTS

A total of 22 THs (14 female; 8 male) were interviewed, along with 16 PLWH (10 female; 6 male). Four themes emerged from the thematic analysis: (1) PLWH prefer THs over HIV clinic workers because they perceive the clinic as not ideal for disclosure; (2) due to the stigma and potential risks of domestic violence or status exposure associated with disclosing their HIV status to family, many PLWH choose to seek support from THs instead; (3) PLWH may choose to voluntarily disclose their status to THs because they are easy to disclose to; and (4) PLWH disclose to their spouses/family members after THs provide guidance on how to do so safely and effectively. These themes emphasize why PLWH prefer THs over formal healthcare workers for disclosure, the impact of stigma and the significant role THs play in encouraging further disclosure to family members.

### 3.1 Preference for THs over formal health workers

PLWH frequently expressed a preference for THs instead of healthcare workers at HIV clinics due to increased trust, confidentiality and the personalized care provided by THs. Participants described healthcare workers in formal settings as often unapproachable, rude or discouraging. Some PLWH noted the important differences between healers and HIV clinic workers, emphasizing that healer environments were less crowded and rushed, which created an environment where healers could be more supportive and helpful.

*Sometimes you are forced to go to the traditional healer so that he or she can help you. Health care providers do not really care about patients at the facility. They are rude and at the same time they do not keep secrets, unlike the traditional healers.—Female, 23, PLWH, baseline interview*

*Services delivered by traditional healers are good. First, they don't delay their clients when they are serving them, there are no big numbers of clients and lines. Secondly, they don't have bad attitudes towards their clients. But health workers, they have bad attitude. They are not good at all. They are so discouraging. Sometimes when they are serving you, you feel like running away and not going back to them.—Female, 25, PLWH, baseline interview*

This trusting environment created a safe space for PLWH to disclose HIV status information. Healers themselves also acknowledge that it is easier for PLWH to disclose to them rather than healthcare workers.

*People in villages do not usually disclose their secrets to formal health workers in health facility. They are feared, not friendly and very busy. They (PLWH) rather disclose to me than health care workers, usually and it becomes easy for them to disclose to tell us [healers].—Female, 60, Traditional healer, baseline interview*

### 3.2 Role of stigma and domestic violence in hindering disclosure

HIV‐related stigma, discrimination and fear of domestic violence emerged as significant barriers to serostatus disclosure within families and communities. Many PLWH shared experiences of social exclusion or anticipated hostility even within their own families.

*Usually, people [in your family] are always jealous of one another and do not wish each other the best. The moment you disclose to any of them, it means the whole world will get to know. The moment people get to know your HIV status they will harass you, discriminate, isolate, or even torture you.—Male, 40, PLWH, baseline interview*

*I have not yet disclosed my HIV status to my parents because I do not what them to know and expose me to my HIV status to the entire public and I become something that everyone talks about.—Female, 20, PLWH, baseline interview*

Furthermore, the fear of domestic violence was particularly noticeable among female participants, many of whom delayed disclosure due to anticipated violence or abandonment.

*She refused to start ARVs because she was fearing her husband to know her status at that moment.—Female, 51, Traditional healer, baseline interview*

*[Some PLWH] lack support from their families and usually also have other issues that are preventing them from staying in care. For instance, the people who have issues of domestic violence cannot stay in care because of failure to disclose to their partners.—Female, 50, Traditional healer, baseline interview*

### 3.3 THs as the first point of disclosure

THs allowed for secrecy from potentially hostile family environments, while facilitating disclosure to the healers also provided much‐needed social support. For many participants, THs were the first individuals to whom they disclosed their status.

*So, whenever [PLWH come to my practice], I first ask about their HIV status depending on their health conditions and in the process, they easily disclose to me and tell me each and everything regarding their HIV status. They would tell me the reasons they defaulted and the challenges they go through while taking HIV medication.—Female, 40, Traditional healer, baseline interview*

This choice was often motivated by the need for someone trustworthy and supportive during moments of vulnerability. One PLWH stated,

*It is not easy to be supported when you have challenges if you have not disclosed your HIV status to anyone. Personally, that is why I disclosed to my traditional healers so that in case I get a challenge, he can always come to my rescue. I had always wanted a close person that I can easily lean on when I am in a critical condition.—Male, 20, PLWH, baseline interview*

THs also played a proactive role in facilitating disclosure, often initiating conversations about health, and creating a safe environment for disclosure. One TH stated,

*It took me a lot of effort to make her speak and slowly with time she was finally able to speak out. She disclosed to me her HIV status. Whenever she came to me while I was serving other people, I would pull her aside and tell her to wait so that we can discuss when I am not so busy—so that I can give her the time and attention that she deserved. That is how she was able to tell me everything and disclose to me that she was HIV infected. But after she disclosed to me, she asked me to make sure I keep her HIV status a secret and confidential—Female, 40, Traditional healer, baseline interview*

### 3.4 Facilitating disclosure to family members

We also noted that healers were sometimes actively involved in supporting PLWH to disclose their HIV status to family members.

*I disclosed to my family members because I wanted them to support me, remind and be there for me when I really needed them. Maybe I would not have disclosed to them, but my healer advised me to do so. He told me that disclosing to my family, will help me to live longer because they will also support me.—Male, 25, PLWH, baseline interview*

*One of my clients who was supposed to go to the facility to get initiated on HIV medication got challenged because she had not disclosed to her mother*. *She was afraid and was not willing to even go to the health facility alone … we discussed disclosure issues and she agreed that she should disclose to her mother. The mother was supportive and agreed to give her psychosocial support, to keep reminding her take her HIV medication on time.—Female, 50, Traditional healer, 3‐month interview*

TH efforts often extended to more direct involvement, such as visiting families or mediating difficult conversations regarding status disclosure.

*There is a woman who has been my client for a long time whom I later discovered that she was hiding her medicine at a neighbor's home because she never wanted her husband to discover that she was on HIV medicine. In the end she ended up defaulting [from care]. But on the other hand, the husband was also in care, he was drinking alcohol a lot … I suggested to disclose to her husband, and that I counsel them together. She accepted, I went to their home and the man was surprised to see me at his home.—Male, 26, Traditional healer, 3‐month interview*

*I did not know how to tell my mother but when I told her [traditional healer] to help me, she did it without hesitating. [The healer] came to our home and told my mother about my condition … I felt ashamed but the good thing is that my mama did not become hostile. The healers counseled both of us well.—Female, 19, PLWH, baseline interview*

## 4 DISCUSSION

Our data highlight the unexpected but important role that THs play in HIV serostatus disclosure among PLWH who are out of clinical care. Currently, status disclosure is hindered by fears of stigma and privacy concerns, especially in healthcare settings []. Our research suggests that THs, who are trusted and accessible community‐based informal healthcare workers, can play a significant role in addressing these challenges [21]. As part of this effort, we explored how THs facilitate serostatus disclosure among PLWH, providing insights on their potential to address critical barriers to HIV care in rural Ugandan communities.

According to the HIV Positive Serostatus Disclosure Model [26, 27], an ideal context for status disclosure should address the following topics with the PLWH: (1) acceptance and empowerment; (2) education and preparation to disclose; (3) establishing motivation for disclosure; (4) evaluation of the disclosure environment; (5) assessing potential outcomes of disclosure; and (6) the disclosure plan and event. Our data suggest that THs can provide this ideal context for disclosure. THs are perceived as trustworthy, caring providers. PLWH and THs have both described that the time, attention and quiet space that is provided makes it easier to facilitate disclosure. Their practices are non‐stigmatized spaces [21, 30], where individuals with many different health concerns seek care. At their practices, healers can explain the importance of status disclosure and support PLWH in their disclosure process, providing them with adequate time to understand their diagnosis.

It is clear that HIV clinics do not currently provide an optimal setting for serostatus disclosure due to perceived lack of confidentiality and trustworthiness, among other factors. However, alternative, non‐clinical spaces for disclosure have not been incorporated into HIV care pathways. One study trained peer educators in Mozambique to give disclosure support for PLWH, which included addressing who the PLWH should tell, how to tell them and giving emotional support [31]. Additionally, religious leaders, as trusted members of their communities, have demonstrated a willingness to speak out about HIV‐related issues. Studies have shown that PLWH are often willing to disclose their status to them; however, these leaders require additional training to effectively provide HIV support and facilitate referrals to appropriate care services [32, 33, 34]. Moreover, a rapid systematic review indicated that differentiated service delivery (DSD) models in African settings achieved retention in care and viral suppression rates comparable to conventional care models [35]. This suggests that DSD approaches may potentially offer more patient‐centred care without compromising clinical outcomes.

Furthermore, the negative interpersonal experiences in formal health facilities may deter disclosure and engagement with care services. These findings underscore the importance of interpersonal trust and cultural competency in supporting PLWH and suggest that traditional healing and patient‐centred approaches could better foster an ideal context for disclosure. THs possess both the cultural sensitivity and interpersonal skills necessary to navigate PLWH and complex family dynamics. The willingness of THs to engage directly with families demonstrates their potential to take on the role of social support people.

The findings highlight an opportunity to integrate THs into HIV care strategies, particularly in settings where stigma and health system barriers hinder effective engagement and disclosure. Training programmes that equip THs with accurate HIV knowledge and skills to provide psychosocial support and referrals could enhance care outcomes on a larger scale [36, 37]. Furthermore, fostering collaborations between THs and formal healthcare providers may address existing gaps in trust and accessibility, ultimately promoting adherence and reducing stigma. We noted that gendered dynamics played a prominent role in shaping disclosure experiences among women, who described fears of intimate partner violence and social vulnerability. These data are consistent with the literature showing that gender‐based power imbalances compound stigma‐related barriers to disclosure among women [38, 39]. Healers in our study were trained to refer participants reporting domestic violence to the social services in their communities, but did not have any specific strategies to mitigate these vulnerabilities among their clients. The risk of intimate partner violence remains a significant challenge for women living with HIV.

These findings have implications beyond the Ugandan study context. As DSD models gain traction across African settings, the insights from our study suggest that community‐based, culturally grounded actors like THs can be meaningfully incorporated into HIV service innovations. In settings where formal health systems are overstretched and stigma remains high, THs may serve as valuable intermediaries who provide psychosocial support, encourage disclosure and facilitate linkage to care. Lessons from this study can inform the design of DSD models to better meet the needs of PLWH who are disengaged from care or facing disclosure‐related barriers, particularly by incorporating informal providers who already hold community trust and deliver personalized, stigma‐free care.

This study has several limitations. First, participants may have responded in socially desirable ways, especially given the healer‐client relationship and the context of participating in an intervention. However, our findings align with other studies among PLWH suggesting that THs are highly trusted providers within their communities [21, 40, 41]. Second, only PLWH who received care from THs were interviewed, which may bias findings towards more favourable perceptions of healers and does not necessarily reflect the contributions that THs may have for serostatus disclosure among PLWH who do not receive traditional care. In addition, although the study suggests potential benefits of integrating THs into HIV care systems, no efficacy data are yet available, underscoring the need for further research to assess longitudinal clinical HIV outcomes, as well as the safety and training needs associated with involving TH in facilitating serostatus disclosure.

## 5 CONCLUSIONS

This study underscores the critical role of THs in facilitating HIV status disclosure by providing an ideal environment and addressing barriers related to stigma and trust. By acknowledging and integrating the contributions of THs, health systems may better support PLWH, particularly in resource‐limited settings where cultural and systemic barriers persist. While our findings suggest that THs play a valuable role in facilitating HIV serostatus disclosure, further research is needed to assess the safety, training requirements and impact of formal integration on clinical outcomes. Integration efforts should be pursued cautiously, with robust implementation research to guide best practices. In addition, future research should explore strategies to formalize these collaborations, ensuring that PLWH have an environment to safely disclose HIV status and receive support through their HIV care itinerary.

## COMPETING INTERESTS

The authors declare no competing interests.

## AUTHORS’ CONTRIBUTIONS

RS designed this study. GN and SG oversaw data collection. CB, WB and MT conducted the interviews with participants. SG, MH and GN analysed the data with supervision from RS. SG was primarily responsible for the writing of the first draft of the manuscript. All authors reviewed the draft, provided input and approved the final version. All authors confirm access to the data in the study and accept responsibility to submit for publication.

## FUNDING

The study was funded by the US National Institute of Mental Health, National Institutes of Health (R01 MH132440).

## Data Availability

The data that support the findings of this study are available from the corresponding author upon reasonable request.
